# Association of Longitudinal Changes in Cerebral Microstructure with Cognitive Functioning in Breast Cancer Survivors after Adjuvant Chemotherapy

**DOI:** 10.3390/jcm13030668

**Published:** 2024-01-24

**Authors:** Vincent Chin-Hung Chen, Yi-Fang Wu, Yuan-Hsiung Tsai, Jun-Cheng Weng

**Affiliations:** 1 School of Medicine, Chang Gung University, Taoyuan 333, Taiwan; 2 Department of Psychiatry, Chang Gung Memorial Hospital, Chiayi 613, Taiwan; 3 Department of Medical Imaging and Radiological Sciences, Chang Gung University, No. 259, Wenhua 1st Rd., Guishan Dist., Taoyuan 333, Taiwan; 4 Department of Diagnostic Radiology, Chang Gung Memorial Hospital, Chiayi 613, Taiwan; 5 Department of Artificial Intelligence, Chang Gung University, Taoyuan 333, Taiwan

**Keywords:** breast cancer, chemotherapy, cognitive impairment, voxel-based morphometry (VBM), vertex-based shape analysis

## Abstract

**Background:** Adjuvant chemotherapy for breast cancer might impact cognitive function and brain structure. **Methods:** In this study, we investigated the cerebral microstructural changes in breast cancer survivors after adjuvant chemotherapy and the correlation with cognitive function with both cross-sectional and longitudinal study designs. All participants underwent structural MRI. In total, we recruited 67 prechemotherapy patients (BB), 67 postchemotherapy patients (BA), and 77 healthy controls (BH). For the follow-up study, 28 participants in the BH and 28 in the BB groups returned for imaging and assessment (BHF, BBF). Voxel-based morphometry analysis was performed to evaluate differences in brain volume; vertex-based shape analysis was used to assess the shape alterations of subcortical regions. Moreover, multiple regression was applied to assess the association between the changes in neuropsychological assessment and brain volume. **Results:** The results showed brain volume reduction in the temporal and parietal gyrus in BB and BA patients. Among each group, we also found significant shape alterations in the caudate and thalamus. Volume reductions in the temporal regions and shape changes in the caudate and hippocampus were also observed in patients from time point 1 to time point 2 (postchemotherapy). An association between brain volume and cognitive performance was also found in the limbic system. **Conclusions:** Based on our findings, we can provide a better understanding of the cerebral structural changes in breast cancer survivors, establish a subsequent prediction model, and serve as a reference for subsequent treatment.

## 1. Introduction

The most common cancer diagnosis among women and the most common cause of death from cancer among women globally is breast cancer. Chemotherapy is the most commonly used treatment method for cancer. Moreover, a significant proportion of cancer patients with chemotherapy experience cognitive dysfunction, which is also known as chemotherapy-induced cognitive impairment (CICI), which results in poor quality of life for these patients. Approximately 75% of chemotherapy patients experience cognitive impairment, and approximately 35% experience cognitive impairment even after completing treatment [1]. Common cognitive problems include difficulties with working memory, attention, executive function, and processing speed [2,3,4,5]. Neuroimaging methods have been used to examine the potential correlation of cognitive impairment in cancer patients. In previous structural MRI studies, they revealed decreased white matter integrity in frontal, parietal, and temporal regions and smaller total brain volume and focal brain volume in breast cancer survivors after receiving chemotherapy [6,7,8].

Previous studies have focused on the relationship between chemotherapy and brain changes or cognitive alterations. Several studies have indicated that cognitive decline may exist even before the start of chemotherapy in the cancer population. An earlier longitudinal study indicated that over 30% of breast cancer patients suffer significant cognitive decline before they undergo treatment [2]. In another longitudinal study, several breast cancer patients diagnosed at stage I–IIIC showed cognitive impairment before undergoing chemotherapy, contributing to problems with memory, attention, and executive functioning [9]. Additionally, stage I–III breast cancer patients have worse cognitive function before treatment than those with stage 0 cancer, as well as healthy controls [10]. It has been reported that there is a relationship between anxiety and depression and impaired cognitive function prior to and after chemotherapy for breast cancer [11]. According to these findings, cognitive impairment may also be influenced by mood symptoms rather than chemotherapy alone [12,13,14,15]. Patients with breast cancer who develop cognitive problems before and after chemotherapy indicate that this is a major concern.

Most recent studies use neuropsychological tests to investigate whether chemotherapy or psychological symptoms affect breast cancer survivors’ cognitive function. To unravel the microstructural brain alterations in the breast cancer population, we adopted voxel-based morphometry (VBM) and vertex-based shape analysis. VBM is an objective approach for estimating the local amount of white matter or gray matter volume changes voxel-by-voxel, and it makes use of a statistical technique for identifying focal differences in brain [16]. However, the shape may change earlier than the volume. Contrary to VBM, vertex-based shape analysis is capable of detecting local appearance differences in subcortical structures [17]. When these two approaches are combined, microstructural changes will be more accurate and detectable.

In this study, we used a cross-sectional and longitudinal study design to investigate brain structural changes in breast cancer survivors. The aim of this study was to explore (1) whether brain volume and shape were altered among patients who received chemotherapy and healthy controls at time point 1; (2) whether any changes in brain volume and shape were observed between time point 1 and 2 in breast cancer patients; and (3) whether neuropsychological performance was associated with brain volume changes in patients with breast cancer. In this study, it was hypothesized that breast cancer survivors treated with and without chemotherapy would have smaller brain volumes in the temporal regions and altered brain shapes of the limbic system compared with healthy controls at time point 1. In addition, brain volume and shape would change from pre- to postchemotherapy in breast cancer survivors, and these changes might indicate cognitive decline.

## 2. Materials and Methods

### 2.1. Participants

In our study, a total of 211 women were recruited from the Chiayi Chang Gung Memorial Hospital, of whom 67 patients with breast cancer did not receive chemotherapy (BB), 67 patients received chemotherapy (BA), and 77 healthy controls (BH). Twenty-eight participants in the BB group returned for assessment after their completion of chemotherapy (BBF). Moreover, 28 participants in the BH group underwent brain MRI at matched intervals as the BBF group (BHF). Breast cancer survivors without brain metastases and older than 20 years of age were included as inclusion criteria. Breast cancer survivors were excluded if they received hormone therapy or other treatment, abused substances, had contraindications for MRI scans, had brain metastasis or brain injuries, or had another form of cancer, as well as cognitive or psychological disorders. Exclusion criteria were the same as in the BH group; the only difference was that they had no history of breast cancer or chemotherapy exposure. This study was approved by the Institutional Review Board of Chang Gung Memorial Hospital, Chiayi, Taiwan. (Nos. 104-5082B, 201700256B0, and 201702027B0). All methods were carried out in accordance with relevant guidelines and regulations. Written informed consent was obtained from all participants.

Each participant completed the Patient Health Questionnaire-9 (PHQ-9) to evaluate depression and the Hospital Anxiety and Depression Scale-Anxiety (HADS-A) to evaluate anxiety. For the longitudinal analysis, measures used in the study included MRI scans of the brain and cognitive assessments. These were completed after the patients were recruited (time point 1) and after they completed chemotherapy. An average time interval of 6-7 months (time point 2) followed the completion of chemotherapy. The healthy controls were also evaluated at the matched time interval.

### 2.2. Cognitive Assessment

For the longitudinal analysis, BB and BBF groups conducted the following assessments. The first part of the Color Trails test (CCT1) was used to evaluate sustained and divided attention; the second part of the Color Trails test (CCT2) was used to evaluate cognitive flexibility. The digit symbol substitution subtest (DSS) of the Wechsler Adult Intelligence Scale—Third Edition was used to assess processing speed. The FACT-Cog was used to evaluate subjective cognitive functioning, with four subscales: perceived cognitive impairment (PCI), perceived cognitive abilities (PCA), impact of perceived cognitive impairment on quality of life (QOL), and comments from others (Oth).

### 2.3. Brain MRI Acquisition

In all participants, the T1-weighted images were acquired on a 3 T MRI scanner (Verio, Siemens, Germany) at Chiayi Chang Gung Memorial Hospital. Brain MRI was performed in the magnetization prepared rapid gradient echo (MPRAGE) sequence with the following parameters: TR/TE = 3500/2.87 ms, TI = 1100 ms, flip angle (FA) = 9 degrees, NEX = 1, field of view (FOV) = 220 × 220 mm^2^, slice thickness = 1 mm, matrix size = 256 × 256, voxel size = 0.9 × 0.9 × 1 mm^3^, and duration = 7.11 min. Additional turbo spin-echo T2 weighted imaging (TSE T2WI) was also performed with the following parameters: TR/TE = 3300/86 ms, flip angle (FA) = 150 degrees, NEX = 1, field of view (FOV) = 220 × 220 mm^2^, slice thickness = 4 mm, matrix size = 256 × 256, slice = 28, and voxel size = 0.9 × 0.9 × 4 mm^3^. The MRI scanner and acquisition parameters were the same between time point 1 and time point 2.

### 2.4. Image Preprocessing

In VBM analysis, images were first preprocessed by the Voxel-Based Morphometry 8 toolbox (VBM, University of Jena, Department of Psychiatry, Jena, Germany) under the Statistical Parametric Mapping (SPM, Wellcome Department of Cognitive Neurology, London, UK). Image motion artifact, distortion, and intensity non-uniformity were first checked. The skull was removed from each image. In the template-based normalization, scans were registered to the East Asian Brain template from the International Consortium for Brain Mapping and then segmented into gray matter, white matter, and cerebrospinal fluid. The segmented tissue maps were modulated to correct the volume change and smoothed by the Gaussian kernel (FWHM = 3 mm) to increase the signal-to-noise ratio. Then the voxel-wise analysis was performed to compare each group using SPM and VBM toolbox.

In the vertex-based shape analysis, images were preprocessed first by the FMRIB Software Library (FSL 5.0). The image artifacts, distortion, and intensity inhomogeneity were checked. The images first removed the skull and then used the FIRST algorithm (FSL-integrated registration and segmentation toolbox) in the FSL to normalize and segment the images to 15 subcortical brain regions, including the brainstem, bilateral hippocampus, bilateral amygdala, bilateral caudate, bilateral putamen, bilateral pallidum, bilateral accumbens, and bilateral thalamus. Non-linear registration (FMRIB’s Non-linear Image Registration Tool, FNIRT) was used to refine the alignment and capture non-linear deformations. This helps in accounting for subtle differences in brain morphology between individuals. We then generated a mesh representation for the 15 brain structures and subsequently aligned each individual mesh with a template mesh. We determined local shape metrics by evaluating shape parameters at every vertex or point on the mesh, such as surface area, curvature, and deformation. Following that, we conducted group-wise statistical comparisons at the vertex level to evaluate variations in shape.

### 2.5. Statistical Analysis

In the cross-sectional analysis, statistics of clinical information were carried out by SPSS. At time point 1, the Kruskal–Wallis test was used to perform the group comparisons in the clinical information. A post hoc Mann–Whitney *U* test was used to evaluate differences between each group.

The brain volume changes among the BB, BA, and BH groups were assessed using the Analysis of Covariance (ANCOVA) model in SPM. Then, post hoc analysis was performed with a two-sample t test to evaluate brain volume changes between each group. The significant results were visualized by xjView software version 10. To evaluate brain shape alteration, we constructed the contrast map in FSL to do the same thing. To avoid original differences in each brain, age, education year, and total intracranial volume were used as covariates. Total intracranial volume was the sum of gray matter, white matter, and CSF. A false discovery rate (FDR)-corrected *p* value of <0.05 was considered to be statistically significant, and was used for every analysis in the study.

In longitudinal analysis, statistics were also performed using SPSS for the neuropsychological tests and patient-reported outcomes. For the patient-reported outcome, two-way mixed-design ANOVA was employed to evaluate group-by-time interactions in the healthy controls and prechemotherapy survivors from time point 1 to time point 2. For the neuropsychological tests, the Wilcoxon signed-rank test was used to evaluate the changes in neuropsychological performance between the BB and BBF groups.

For changes in brain volume, we used a two-way repeated measures analysis of variance model in SPM to evaluate the brain volume change between time point 1 and time point 2 in the BB group relative to the BH group. This ANOVA model included the time factor, group factor, and subject factor. The analysis utilized a flexible factorial design in SPM to explore group-by-time interactions. Then, we used the post hoc paired t test to explore the difference within the group from time point 1 to time point 2. For the shape alteration, contrast maps were constructed to do the same thing. Corrected *p* values < 0.05 were considered significant.

In the association analysis, multiple regression was used to investigate the association between neuropsychological performance changes (time point 2 minus time point 1) and brain volume changes (time point 2 minus time point 1) between the BB and BBF groups. The general linear model in SPM was used to find the association. To avoid original differences in each brain, age, education year, and total intracranial volume were entered as covariates. Furthermore, the volume values were extracted from the segmented gray or white matter images for the multiple regression analysis. Corrected *p* values < 0.05 were considered significant.

## 3. Results

### 3.1. Demographic Characteristics

There were 211 female participants recruited from Chiayi Chang Gung Hospital. The demographic characteristics of all participants are summarized in Table 1. In BA groups, 67 patients who received chemotherapy were examined only after having completed chemotherapy. The days after complete chemotherapy were 223 ± 142.9. At time point 1, there were no significant differences among the three groups in terms of total intracranial volume. However, comparison of the BB and BH groups revealed variations in age and education years. There was an assessment of anxiety and depression for all participants. There were no significant differences among BB, BA, and BH groups at time point 1, except for depression. The BB group scored significantly higher than BH on the PHQ-9, but there were no differences between the BA and BB groups or between the BA and BH groups on the PHQ-9.

### 3.2. Neuropsychological Assessment and Patient-Reported Outcomes

Table 2 summarizes the neuropsychological results. Cognitive testing was conducted only on the BB group. Twenty-eight participants in the BB group returned for imaging and assessment in time point 2, and they underwent posttreatment assessments for the paired test. The cognitive performance of the BBF group was worse than that of the BB group. In addition, the FACT-Cog results showed that the BB group had worse cognitive function over time. Table 3 summarizes the patient-reported outcomes. The group-by-time interaction showed significant differences only for the HADS-A scale. There was a significant difference in HADS-A scores between the BB and BBF groups.

### 3.3. Cross-Sectional Analysis

**Figure 1:**
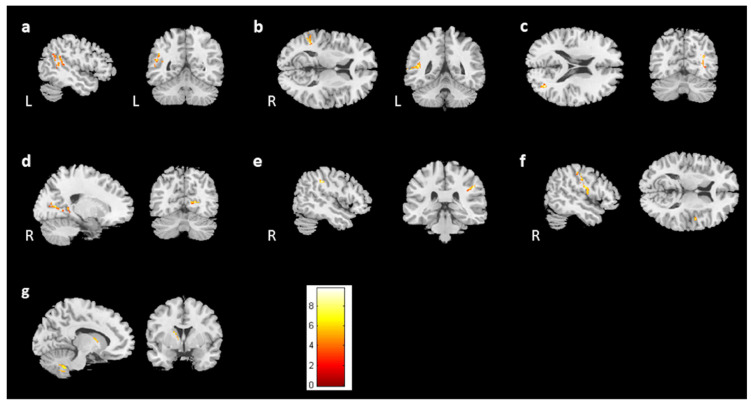
Results of ANCOVA in the BB, BA, and BH groups. Among the BB, BA, and BH groups, we found significant differences between (**a**) the left angular gyrus, (**b**,**c**) bilateral middle temporal gyrus, (**d**) right calcarine, (**e**) right postcentral gyrus, (**f**) right Rolandic operculum, and (**g**) left caudate. (corrected *p* value < 0.04, cluster size > 150, color bar: F scores).

**Figure 2:**
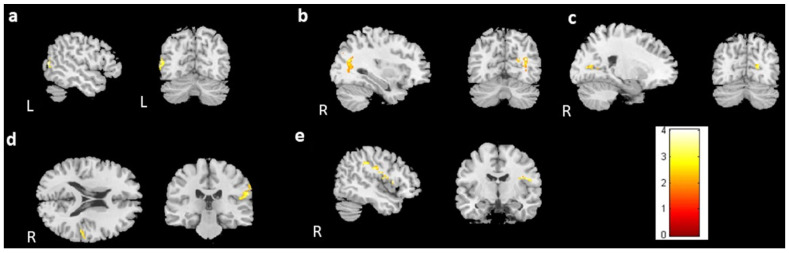
Post hoc *t* test results between the BB and BA groups. Significantly larger volumes of the (**a**) left middle temporal gyrus, (**b**) right middle temporal gyrus, (**c**) right calcarine, (**d**) right postcentral gyrus, and (**e**) right Rolandic operculum were observed in the BB group than in the BA group. (BB > BA, corrected *p* value < 0.03, cluster size > 150, color bar: T scores).

**Figure 3:**
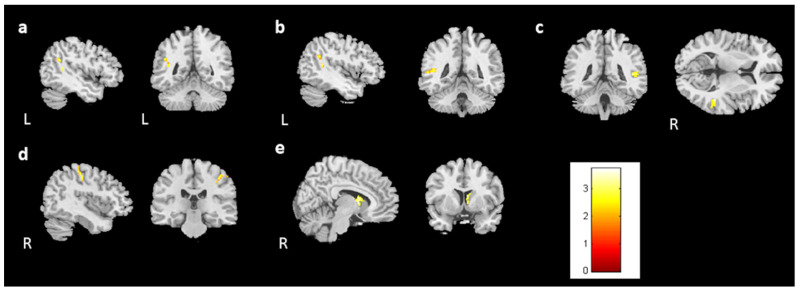
Post hoc *t* test results between the BH and BB groups. Significantly larger volumes of the (**a**) left angular gyrus, (**b**) left superior temporal gyrus, (**c**) right middle temporal gyrus, (**d**) right postcentral gyrus, and (**e**) right caudate were observed in the BH group than in the BB group. (BH > BB, corrected *p* value < 0.03, cluster size > 150, color bar: T scores).

**Figure 4:**
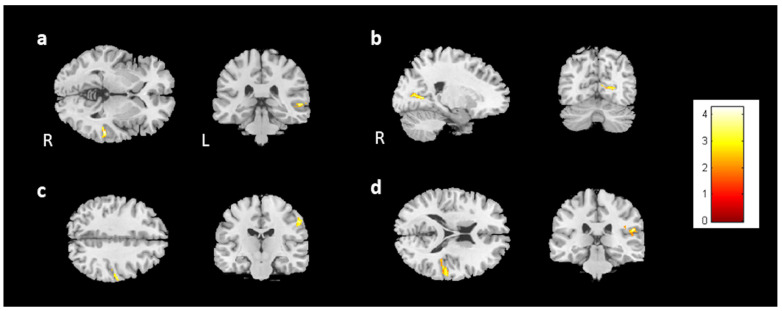
Post hoc *t* test between the BH and BA groups. Significantly larger volumes of the (**a**) right middle temporal gyrus, (**b**) right calcarine, (**c**) right postcentral gyrus, and (**d**) right Rolandic operculum were observed in the BH group than in the BA group. (BH > BA, corrected *p* value < 0.03, cluster size > 150, color bar: T scores).

For the cross-sectional study, 67 prechemotherapy patients (BB), 67 postchemotherapy patients (BA), and 77 healthy controls (BH) were recruited. In the voxel-based morphometry, volume differences among the BB, BA, and BH groups were evaluated using ANCOVA. We found significant differences in brain volume in the left angular gyrus, bilateral middle temporal gyrus, right calcarine, right postcentral gyrus, right Rolandic operculum, and left caudate (Figure 1, corrected *p* value < 0.04, cluster size > 150). To further evaluate the differences between each group, a two-sample t test was conducted as a post hoc test. The BA group had significantly lower brain volumes than the BB group in the bilateral temporal gyrus, right postcentral gyrus, right Rolandic operculum, and right calcarine (Figure 2, corrected *p* value < 0.03, cluster size > 150). In comparison to the BH group, both the BB and BA groups had significantly lower volumes in the right middle temporal gyrus and right postcentral gyrus (Figure 3c,d and Figure 4a,c). For Figure 3 and Figure 4, corrected *p* value < 0.03, cluster size > 150. There was a significant difference between the BA and BH groups for the right Rolandic operculum and right calcarine (Figure 4b,d). Furthermore, in the left angular gyrus, left superior temporal gyrus, and right caudate, the results indicated lower brain volume in the BB group than in the BH group (Figure 3a,b,e). A summary of the cross-sectional findings in the VBM analysis can be found in Table S1.

**Figure 5:**
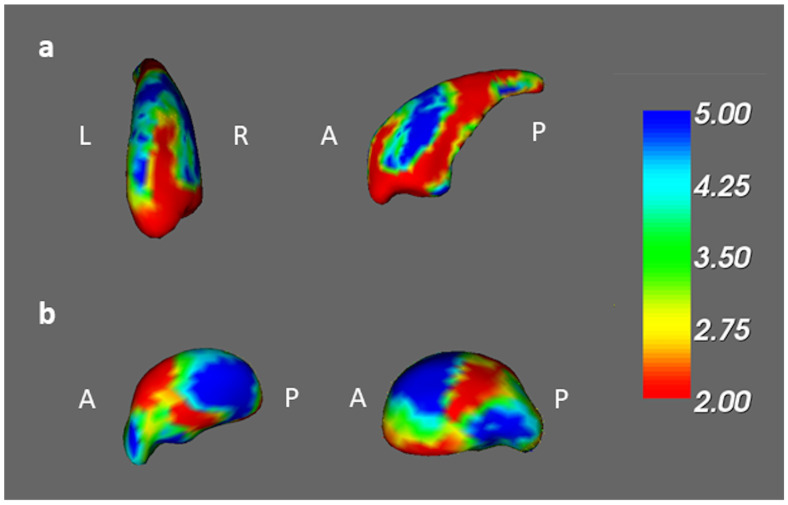
The ANCOVA results among the BB, BA, and BH groups. Among the BB, BA, and BH groups, we found significant shape alterations in the (**a**) right caudate and (**b**) right thalamus (corrected *p* value < 0.05, color bar: F scores, A: anterior, P: posterior).

**Figure 6:**
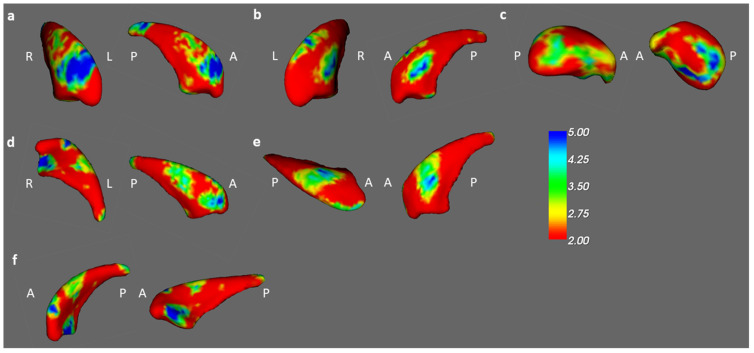
Post hoc *t* test results in the BB, BA, and BH groups. Significant shape differences were found in the ((**a**): left, (**b**): right) bilateral caudate and (**c**) right thalamus between the BB and BA groups. In comparison of the BH and BB groups, the results were observed in the ((**d**): left, (**e**): right) bilateral caudates. Moreover, significant differences between the BH and BA groups were observed in the (**f**) left caudate. (corrected *p* value < 0.05, color bar: F scores, A: anterior, P: posterior).

In the vertex-based shape analysis, the BB, BA, and BH groups differed significantly in the shape of their right caudates and right thalamus (Figure 5). When comparing to BB groups, there was a significant difference in concavity of BA in the bilateral caudates and right thalamus (Figure 6a–c). Compared to BH groups, we found a significant difference in concavity of BB in the bilateral caudate (Figure 6d,e). A similar finding but more pronounced difference also exists between the shapes of the left caudate between BA and BH (Figure 6f).

### 3.4. Longitudinal Analysis

**Figure 7:**
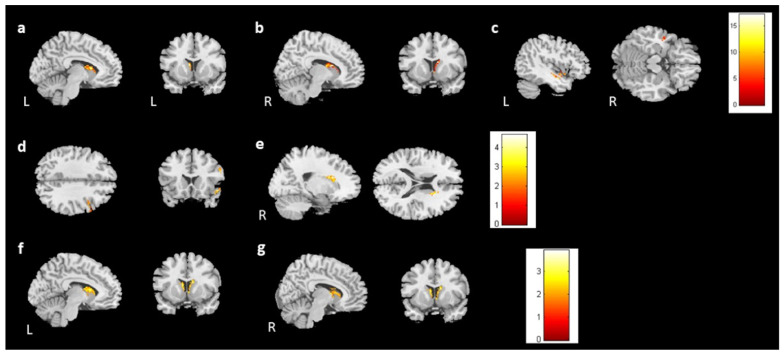
The volume differences in the longitudinal study. There were volume differences in the (**a**) left caudate, (**b**) right caudate, and (**c**) left middle temporal gyrus between the prechemotherapy patients and healthy controls from TP1 to TP2. (*p* value < 0.05, cluster size > 150, color bar: F scores) In the paired t test, there were volume differences in the (**d**) right superior temporal gyrus (BBF < BB) and (**e**) right caudate between the BB and BBF groups (BBF > BB). Moreover, volume reductions in the bilateral caudates ((**f**): left; (**g**): right) were observed between the BH and BHF groups. (BHF < BH, corrected *p* value < 0.05, cluster size > 150, color bar: T scores).

For the follow-up study, 28 participants in the BH and 28 in the BB groups returned for imaging and assessment (BHF, BBF). In the voxel-based morphometry (group-by-time interaction), the bilateral caudate and left middle temporal gyrus showed differing brain volumes between healthy control and prechemotherapy patients from time point 1 to time point 2 (Figure 7a–c). Furthermore, the right superior temporal gyrus volumes were reduced in the BBF group compared with the BB group (Figure 7d). However, the BBF group had a greater volume increase in the right caudate (Figure 7e). Additionally, we found that the bilateral caudate volume was reduced in the BHF group compared to the BH group (Figure 7f,g). The summary of longitudinal findings in VBM analysis can be found in Table S2. For Figure 7, corrected *p* value < 0.05, cluster size > 150.

**Figure 8:**
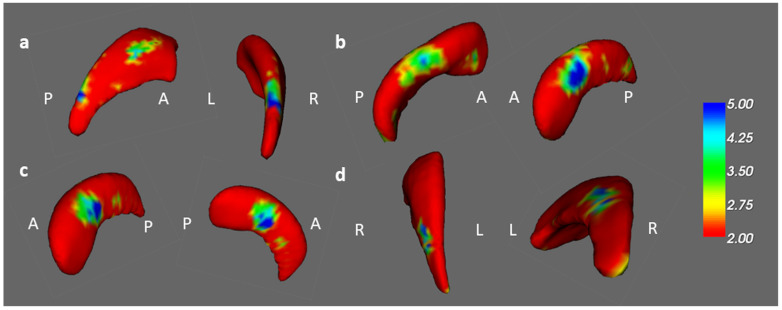
The shape result in the longitudinal study. There were significant shape changes in the (**a**) left caudate and (**b**) left hippocampus between the prechemotherapy patients and healthy controls from TP1 to TP2. (corrected *p* value < 0.05, color bar: F scores) In the paired t test, we observed shape alterations in the (**c**) left hippocampus between the BB and BBF groups and in the (**d**) right caudate between the BH and BHF groups. (corrected *p* value < 0.05, color bar: F scores, A: anterior, P: posterior).

In the vertex-based shape analysis, a comparison of the healthy controls and prechemotherapy patients revealed differences in caudate and hippocampal shapes on the left side (Figure 8a,b). Additionally, paired t tests revealed a difference in the shape of the left hippocampus between the BB and BBF groups (Figure 8c). A difference in the shape of the right caudate was found between the BH and BHF groups (Figure 8d).

### 3.5. Association Analysis

**Figure 9:**
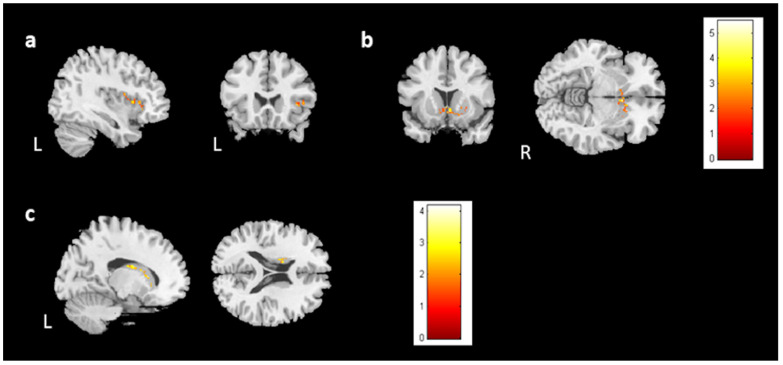
The results of the association between brain volume and the HADS-A/DSS. The results showed a negative association between the HADS-A and brain volume in the (**a**) right insula and (**b**) bilateral caudate. Moreover, a positive association was observed between the DSS and brain volume in the (**c**) left caudate. (corrected *p* value < 0.05, cluster size > 150, color bar: T scores).

We analyzed the association between brain volume change and cognitive performance or mood symptoms (change scores) for 28 participants from the BB and BBF groups (Supplementary Figure S1). One outlier was ruled out in the analysis. In the analysis, the HADS-A was negatively associated with gray matter volume changes in the right insula and bilateral caudate (Figure 9a,b). The analysis did not find a significant association between brain volume change and PHQ-9.

**Figure 10:**
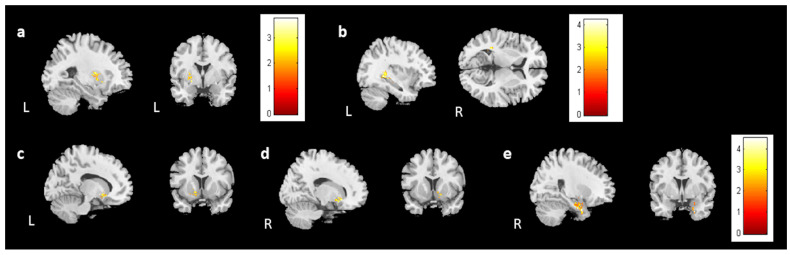
The results of the association between brain volume and FACT-Cog scores. A positive association was found between (**a**) the FACT-Cog scores and the brain volume in the left putamen, (**b**) the PCI subtest and the volume of the left temporal gyrus, and (**c**–**e**) the PCA subtest and the volume of the bilateral putamen and right parahippocampus. (corrected *p* value < 0.05, cluster size > 150, color bar: T scores).

For the evaluations of cognitive performance, a decrease in DSS scores was associated with reduced volume in the left caudate (Figure 9c). CTT1 and CTT2 did not show any significant association with brain volume changes. In subjective cognitive assessments, there was an association between decreased FACT-Cog and reduced volume in the left putamen (Figure 10a). Furthermore, the analysis identified a positive association between perceived cognitive impairment and volume changes in the left temporal gyrus (Figure 10b). Perceived cognitive abilities were also positively associated with volume changes in the bilateral putamen and left parahippocampus (Figure 10c–e). For Figure 9 and Figure 10, corrected *p* value < 0.05, cluster size > 150.

## 4. Discussion

In this study, brain volume and shape differences were investigated cross-sectionally and longitudinally in patients with breast cancer, as well as the interaction of brain volume changes with cognitive performance. In cross-sectional analysis, the caudate, right postcentral gyrus, and temporal gyri have been observed to have changed in volume and shape. In the longitudinal studies, we observed changes in the caudate and temporal areas. In addition, association analysis revealed a significant correlation between certain brain volumes and cognitive function.

### 4.1. Prechemotherapy Difference

Study findings showed that brain volume and shape were altered in women without chemotherapy for breast cancer. In our results, lower white matter volume was found in the left superior temporal gyrus, and lower gray matter volume was found in the right middle temporal gyrus and postcentral gyrus. The temporal region has played a role in a variety of cognitive processes. It has been shown that the middle temporal gyrus participates in language processing [18], grasp observation [19], and deductive reasoning [20]. Studies have shown that the middle temporal region is associated with the default mode network (DMN) and semantic memory network (SMN). Additionally, the superior temporal gyrus is involved in auditory processing, social cognition, and spatial awareness [21]. It has been reported that a decrease in the volume of the temporal regions may indicate a decrease in the number of neurons, which may lead to reading difficulties [22].

The affected brain regions in this study were generally in accordance with those observed in previous studies of cancer populations. They reported reduced fractional anisotropy in temporal white matter tracts in a diffusion tensor study [6]. A study of structural brain differences in non-CNS cancer patients, moreover, found significant reductions in cortical surface area or thickness in the temporal and parietal regions [23]. Our study also found that the caudate had lower volume and a change in shape. Previously, researchers explored whether certain brain regions play a role in learning and memory. They reported that depressed participants have smaller volumes of the anterior caudate, indicating that the caudate plays a role in learning and forming memories [24]. Furthermore, a resting-state fMRI study reported that caudate hypoactivity is associated with depression in breast cancer patients before receiving chemotherapy [25].

Compared with previous studies, our study also identified volume alterations of the brain that had not been previously reported. Volume reductions were observed in the angular gyrus, a component of the DMN and a connector hub. Researchers have revealed that depressed participants have higher brain activity in this region than those without depression. Consequently, they suggest that disruption of the DMN might be associated with the alteration of the angular gyrus [26]. Moreover, according to previous studies, DMN alterations might be associated with poorer cognitive function [27,28]. We found differences in brain structure between the groups even before chemotherapy began. Although the exact potential mechanism is unknown, the reduced brain volume in the BB group may result from a combination of the disease process and interaction of mood symptoms [29].

### 4.2. Postchemotherapy Differences

According to our study, breast cancer patients receiving chemotherapy had alterations in brain volume and shape. Our cross-sectional analysis found diminished temporal gyri volumes as well as changes in caudate and thalamus shape in chemotherapy-treated patients compared to chemotherapy-untreated patients. Similarly, our longitudinal study found volume changes in the temporal gyri and right caudate from time point 1 to time point 2 (postchemotherapy) in breast cancer patients. This was consistent with previous studies that had similar study designs. A previous study used Free-Surfer software version 6.0.1 in small-cell lung cancer patients and found differences in frontal, temporal, and parietal areas [30]. According to some longitudinal studies, breast cancer survivors experienced changes in brain structure after chemotherapy exposure. Using VBM, McDonald et al. found a significant decrease in gray matter density in bilateral frontal, temporal, and thalamic regions in breast cancer patients from prechemotherapy to postchemotherapy. They suggest that chemotherapy caused the loss of volume, rather than host factors, the underlying cancer process, or another treatment effect. As a result of their findings, we speculated that chemotherapy might be responsible for the volume reduction in these regions we found.

In cross-sectional analysis, we also found a lower volume in the right Rolandic operculum. The Rolandic operculum, or subcentral gyrus, is the inferior part of the postcentral gyrus and precentral gyrus. Previously, resting-state functional magnetic resonance imaging and diffusion tensor imaging were used to explore chemotherapy-related cognitive disorders in Asian breast cancer survivors. The results show that patients who received chemotherapy have lower ReHo in the right subcentral area [31]. Moreover, our longitudinal analysis revealed that the caudate volume increased in breast cancer survivors after receiving chemotherapy, as did changes in hippocampal shape. Two studies have examined gray matter density/volume in women with breast cancer receiving chemotherapy. They found decreased gray matter density/volume in the hippocampus in women with breast cancer one month after chemotherapy completion [32,33]. Survivors still have a reduced hippocampal volume after chemotherapy has been completed for several years. In their study, they suggested that the size of the hippocampus is related to autobiographical memory retrieval and previous cancer experiences [34]. Furthermore, Kesler and colleagues reported that cytokine levels are related to hippocampal volume. It has been reported that doxorubicin elevates tumor necrosis factor-alpha levels, specifically in the hippocampal region of animals [35,36,37]. On the other hand, the increase in caudate volume may be attributed to a compensatory mechanism. It has been shown in a previous study that there is an inverse relationship between the hippocampus and caudate and that enlargement of the caudate compensates for decreased hippocampal activity [38].

Postcentral gyrus volume has not been reported in previous volumetric analyses of breast cancer. In a study of ovarian cancer women, they used VBM analysis to explore the differences between chemotherapy-treated patients and healthy controls and found that frontal and postcentral gray matter volumes were reduced [39]. However, MRI and DTI studies of breast cancer patients have shown less NAA and creatine in the thalamus. The NAA reduction in gray matter is associated with neuron loss and metabolic inactivity, while the NAA reduction in white matter may be related to axonal damage. In addition, total creatine is a marker of cell integrity. It was reported that a reduced creatine level in chemotherapy patients was indicative of intracranial injury, which was likely to result in cognitive deficits [40]. This study explored some brain regions not previously explored in previous studies of women with breast cancer. We found that the volume of the right calcarine was reduced in the BA group compared with the BB group. The primary visual cortex is located here and connected to the visual cortex. VBM was used to investigate volume changes in a childhood leukemia study, which revealed that patients show a decrease in visual processing ability and recognition memory due to a smaller calcarine gyrus volume [41].

Although volume loss and shape changes can be related, they represent distinct aspects of brain structure and are typically assessed separately. Volume loss is a reduction in the overall size or volume of specific brain regions or the entire brain. Shape changes involve alterations in the geometry or morphology of brain structures without necessarily reflecting a change in overall volume. While volume loss and shape changes can occur concurrently, they do not always correlate directly. Our findings showed uniformity in both volume loss and shape changes observed in the BH and BHF groups. However, there were slight variations in these aspects between the BB and BBF groups.

### 4.3. Association between Brain Volume and Cognitive Performance

For the cognitive assessment, the DSS scores decreased when more volume reduction was observed in the left caudate. The caudate is an important component of the frontostriatal loops that control executive function. A lesion in this area affects processing speed, attention, and memory [42]. A study on white matter changes found that caudate volume loss was not associated with the severity of white matter changes but with impaired executive function [43].

Our findings showed an association between decreased FACT-Cog scores and more volume reduction in the left thalamus and left putamen. For the subscales, the decreased PCA scores were associated with decreased volume in the bilateral putamen and right parahippocampus; the PCI scores were associated with diminished volume in the temporal gyrus on the left side. FACT-Cog assessed memory, attention, and the impact of disturbances on patient quality of life. Previous research has found that the PCA and PCI subscales are associated with objective assessments of verbal memory and executive function. In particular, PCA may provide insight into cognitive function in patients with breast cancer [44]. There is evidence that the putamen is involved in the attentional process and affected by chemotherapy in breast cancer survivors [45]. Moreover, reduced parahippocampal volume has been associated with various types of chemotherapy [46]. The parahippocampus is involved in cognitive functions such as memory and visuospatial processing. The putamen is associated with verbal memory encoding. Additionally, the volume of the thalamus and temporal lobes is related to verbal memory performance, meaning larger volumes correlate with better word recall [47]. The results of our study provide evidence that there was some association between brain structure and neuropsychological performance. Future studies with large sample sizes and comprehensive psychological tests will be necessary to investigate the association between brain alterations and cognitive performance in detail.

### 4.4. Possible Clinical Implications

There were several potential clinical implications for our study. First, the observed brain volume reduction in the temporal and parietal gyrus in breast cancer survivors after chemotherapy suggests potential cognitive implications. Clinicians may consider implementing regular cognitive monitoring in breast cancer survivors, especially those who have undergone chemotherapy, to detect and address any cognitive changes early on. Cognitive interventions or rehabilitation programs targeting the affected brain regions may be explored to mitigate cognitive decline and improve the quality of life for breast cancer survivors. Second, the longitudinal aspect of the study, with follow-up imaging and assessments, emphasizes the importance of long-term follow-up care for breast cancer survivors. Clinicians may incorporate regular neuroimaging and cognitive assessments into survivorship care plans to monitor any ongoing changes and address the evolving needs of breast cancer survivors post-treatment. Finally, the association between brain volume and cognitive performance in the limbic system suggests a potential target for interventions. Therapies or interventions aimed at preserving or enhancing limbic system function may be explored to improve cognitive outcomes in breast cancer survivors.

### 4.5. Limitations

There were some potential limitations to our study. First, there were a limited number of participants in the BBF and BHF groups. Therefore, it would make it limited to assessing brain volume and neuropsychological changes. Second, the study is the lack of a cancer group not treated with chemotherapy assessed longitudinally. Third, participants in the BHF group did not conduct the cognitive assessment. To provide more robust results, the cognitive assessments of healthy controls can be included in future studies. Fourth, there were differences in stage distribution between BA and BB groups in the cross-sectional study. However, the results from the cross-sectional study, such as higher anxiety level in the pre-chemotherapy group, was also found in the follow-up study. Further studies can use more homogenous groups to detect this issue. Finally, we did not take fatigue, menopause, different chemotherapy regimens, or general health pre-cancer diagnosis (e.g., co-occurring disorders that could influence brain oxygenation) into account. Future research needs to investigate whether these factors affect the results.

## 5. Conclusions

The purpose of this study was to investigate brain microstructural changes in breast cancer survivors who had received adjuvant chemotherapy. In summary, we found that there were significant findings that indicated that even before chemotherapy, breast cancer survivors had both volume and shape changes to their brains. Both cross-sectional and longitudinal results suggest that chemotherapy may result in a volume reduction in breast cancer survivors. Moreover, we found that the brain volume of certain regions was associated with cognitive performance. These results suggest that structural neuroanatomical correlates may exist and affect cognitive impairment before and after chemotherapy. Despite its limitations, this study had some strengths. We examined the brain microstructure in detail by combining longitudinal and cross-sectional designs. A combination of longitudinal and cross-sectional designs allowed us to examine the brain microstructure in detail. Comparing our findings from the cross-sectional analysis with previous studies, the methodology was accurate. In the longitudinal analysis, the sample size was larger than in the previous studies, which could minimize bias. Furthermore, longitudinal analysis allowed us to understand the relationship between brain volume and cognitive performance in women with breast cancer. To the best of our knowledge, this study was one of the few studies using shape analysis in breast cancer survivors. With the aid of shape analysis, we were able to fill the knowledge gap regarding microstructural brain changes in breast cancer survivors. According to our findings, the study has potential to develop a prediction model and serve as a reference for subsequent treatment.

## Figures and Tables

**Table 1:** Demographic characteristics.

	BA	BB	BH	Kruskal–Wallis Test	A	B	C
	(N = 67)	tp1 (N = 67)	tp1 (N = 77)				
	Mean/No. (SD)	Mean/No. (SD)	Mean (SD)	Corrected *p* Value			
Age at tp1, years	49.1 (8.3)	50.2 (8.8)	47.0 (8)	0.45	0.51	0.02 *	0.09
Education, years	12.3 (3.8)	11.9 (3.9)	13 (3.2)	0.08	0.61	0.03 *	0.11
PHQ-9	3.9 (3.7)	4.7 (4.1)	2.9 (2.9)	0.02 *	0.91	<0.01 *	0.12
HADS-A	3.6 (3.8)	5 (4.4)	3.9 (3.6)	0.06	0.04 *	0.12	0.43
Intracranial volume, milliliter	1261.5 (100.7)	1261.7 (97.8)	1269.8 (95.4)	0.76	0.98	0.48	0.60
days after complete chemotherapy	223 (142.9)						
Breast cancer stage							
0	0	19					
I	13	19					
II	37	21					
III	13	6					
IV	4	2					

Abbreviations: BA, postchemotherapy patients; BB, prechemotherapy patients; BH, healthy controls; tp1, time point 1; PHQ-9, Patient Health Questionnaire; HADS-A, Hospital Anxiety and Depression Scale–Anxiety. A = comparison between breast cancer patients before and after chemotherapy at time point 1 (BA; BB); B = comparison between breast cancer patients before chemotherapy and healthy controls at time point 1 (BB; BH); C = comparison between breast cancer patients after chemotherapy and healthy controls at time point 1 (BA; BH). Asterisks indicate significant differences (*p* < 0.05).

**Table 2:** Summary of the neuropsychological evaluation.

	BB	BBF	
	tp1 (N = 28)	tp2 (N = 28)	Paired Test
	Mean (SD)	Mean (SD)	Corrected *p* Value
Color Trails Test 1 (completion time)	49.51 (16.18)	50.18 (25.77)	0.412
Color Trails Test 2 (completion time)	95.22 (30.82)	99.41 (46.95)	0.802
Digit symbol substitution (raw scores)	63.54 (18.48)	64.11 (19.19)	0.799
FACT-Cog	120.71 (11.45)	115.39 (11.88)	0.015 *
Perceived Cognitive Impairments	67.14 (6.69)	63.61 (7.20)	0.016 *
Perceived Cognitive Abilities	23.04 (4.42)	21.29 (3.61)	0.064
Impact of Perceived Cognitive Impairments on QoL	15.00 (2.28)	14.89 (2.55)	0.554
Comments from Others	15.54 (1.04)	15.61 (0.83)	0.668

Abbreviation: BB, prechemotherapy patients; BBF: patients who returned for posttreatment assessment; tp1, time point 1; tp2, time point 2; FACT-Cog, Functional Assessment of Cancer Therapy Cognitive Scale. Asterisks indicate significant differences (*p* < 0.05).

**Table 3:** Summary of the patient-reported outcome.

	BB	BBF		BH	BHF		Group Time Interaction
	tp1 (N = 28)	tp2 (N = 28)	Paired Test	tp1 (N = 28)	tp2 (N = 28)	Paired Test	
	Mean (SD)	Mean (SD)	Corrected *p* Value	Mean (SD)	Mean (SD)	Corrected *p* Value	
	3.68 (2.72)	2.82 (2.36)	0.06	2.32 (2.48)	2.00 (2.14)	0.483	0.891
HADS-A	3.21 (2.91)	1.35 (1.87)	<0.001 *	2.35 (2.14)	2.57 (2.69)	0.634	<0.01 *

Abbreviation: BB, prechemotherapy patients; BBF: patients who returned for posttreatment assessment; BH, healthy controls; BHF, participants who returned for assessment again; tp1, time point 1; tp2, time point 2; PHQ-9, Patient Health Questionnaire; HADS-A, Hospital Anxiety and Depression Scale–Anxiety. Asterisks indicate significant differences (*p* < 0.05).

## Data Availability

The raw data supporting the conclusions of this article will be made available by the corresponding author on request.

## Supplementary Materials

The following supporting information can be downloaded at: https://www.mdpi.com/article/10.3390/jcm13030668/s1, Figure S1: The scatter plots of the correlation analysis; Table S1: Summary of cross-sectional findings in VBM analysis; Table S2: Summary of longitudinal findings in VBM analysis.
