# Supplementary material for: Association of Longitudinal Changes in Cerebral Microstructure with Cognitive Functioning in Breast Cancer Survivors after Adjuvant Chemotherapy
Source: J Clin Med. 2024 Jan 24;13(3):668. doi: 10.3390/jcm13030668 (PMC10856189; doi:10.3390/jcm13030668)
Supplement: Supplementary file 1 [file jcm-13-00668-s001.zip › Supplementary FigS1.docx]

**Supplementary Figure S1 The scatter plots of the correlation analysis**
