# Supplementary material for: Association of Longitudinal Changes in Cerebral Microstructure with Cognitive Functioning in Breast Cancer Survivors after Adjuvant Chemotherapy
Source: J Clin Med. 2024 Jan 24;13(3):668. doi: 10.3390/jcm13030668 (PMC10856189; doi:10.3390/jcm13030668)
Supplement: Supplementary file 1 [file jcm-13-00668-s001.zip › Supplementary Tables.docx]

**Table S1 Summary of cross-sectional findings in VBM analysis**

| **Region (for cluster peak)** | **Gray or white matter** | **MNI coordinates (x y z)** | **peak F score** | **corr. p value** |
| --- | --- | --- | --- | --- |
| Left angular gyrus | WM | -47 -51 27 | 8.17 | 0.04 |
| Left middle temporal gyrus | WM | -47 -47 13 | 7.06 | 0.04 |
| Right middle temporal gyrus | WM | 36- 72 28 | 11.11 | 0.04 |
| Right calcarine | GM | 20 -66 7 | 9.89 | 0.04 |
| Right postcentral gyrus | GM | 48 -32 38 | 8.8 | 0.04 |
| Right Rolandic operculum | WM | 48 -4 13 | 7.45 | 0.03 |
| Left caudate | WM | -15 -7 19 | 9.61 | 0.04 |
|  |  |  |  |  |
| **Region (for cluster peak)** | **Gray or white matter** | **MNI coordinates (x y z)** | **peak T score** | **corr. p value** |
| *BB > BA* |  |  |  |  |
| Left middle temporal gyrus | WM | -51 -70 9 | 3.37 | 0.03 |
| Right middle temporal gyrus | WM | 35 -67 10 | 4.62 | 0.02 |
| Right calcarine | WM | 24 -60 15 | 4.42 | 0.02 |
| Right postcentral gyrus | WM | 48 -30 37 | 4.1 | 0.02 |
| Right Rolandic operculum | GM | 51 -28 19 | 3.57 | 0.02 |
| *BH > BB* |  |  |  |  |
| Left angular gyrus | WM | -47 -51 27 | 3.94 | 0.03 |
| Left superior temporal gyrus | WM | -45 -45 12 | 3.56 | 0.03 |
| Right middle temporal gyrus | WM | 50 -34- 2 | 3.01 | 0.03 |
| Right postcentral gyrus | GM | 39 -31 59 | 4.03 | 0.03 |
| right caudate | GM | 8 5 4 | 2.81 | 0.03 |
| *BH > BA* |  |  |  |  |
| Right middle temporal gyrus | GM | 55 -31 0 | 4.1 | 0.03 |
| Right calcarine | WM | 24 -60 12 | 4.05 | 0.02 |
| Right postcentral gyrus | WM | 54 -22 49 | 3.52 | 0.02 |
| Right Rolandic operculum | WM | 51 -28 21 | 4.26 | 0.02 |

The coordinates of the results were in the Montreal Neurological Institute (MNI) space. Regions are the differences in brain volume among the BB, BA and BH groups. Abbreviations: BA, postchemotherapy patients; BB, prechemotherapy patients; BH, healthy controls

**Table S2 Summary of longitudinal findings in VBM analysis**

| **Region (for cluster peak)** | **Gray or white matter** | **MNI coordinates (x y z)** | **peak F score** | **corr. p value** |
| --- | --- | --- | --- | --- |
| Left caudate | GM | -8 12 10 | 16.05 | 0.04 |
| Right caudate | GM | 11 11 16 | 20.71 | 0.05 |
| Left middle temporal gyrus | WM | -45 -6 -17 | 17.21 | 0.05 |
|  |  |  |  |  |
| **Region (for cluster peak)** | **Gray or white matter** | **MNI coordinates (x y z)** | **peak T score** | **corr. p value** |
| *BB > BBF* |  |  |  |  |
| Right superior temporal gyrus | GM | 47 17 -12 | 3.22 | 0.05 |
| Right caudate | WM | 20 11 20 | 2.93 | 0.05 |
| *BH > BHF* |  |  | 2.56 |  |
| Left caudate | GM | -8 12 10 | 3.85 | 0.05 |
| Right caudate | GM | 11 12 18 | 3.8 | 0.05 |

The coordinates of the results were in the Montreal Neurological Institute (MNI) space. Regions are the differences in brain volume in longitudinal analysis. Abbreviation: BB, prechemotherapy patients; BBF: patients who returned for posttreatment assessment; BH, healthy controls; BHF, participants who returned for assessment again
